# The Effect of 5-Aza-2′-Deoxycytidine in Combination to and in Comparison with Vorinostat on DNA Methyltransferases, Histone Deacetylase 1, Glutathione S-Transferase 1 and Suppressor of Cytokine Signaling 1 Genes Expression, Cell Growth Inhibition and Apoptotic Induction in Hepatocellular LCL-PI 11 Cell Line

**Published:** 2020-01-01

**Authors:** Masumeh Sanaei, Fraidoon Kavoosi, Zahra Esmi

**Affiliations:** 1 Research Center for Non-communicable Diseases, Jahrom University of Medical Sciences, Jahrom, Iran; 2 Research Committee Student, Jahrom University of Medical Sciences, Jahrom, Iran

**Keywords:** Epi-drugs, Tumor suppressor genes, Cancer

## Abstract

**Background:** Aberrant methylation and histone deacetylation of tumor suppressor genes (TSGs) are the most epigenetic alterations involving in tumorigenesis. Overexpression of DNA methyltransferases (DNMTs) and histone deacetylase 1 (HDAC1) have been reported in several cancers. The reversion of hypermethylation and deacetylation by epi-drugs such as 5-aza-2′-deoxycytidine (5-AZA-CdR) and vorinostat (SAHA) can restore normal expression of TSGs. Previously, we reported that 5-AZA-CdR and valproic acid (VPA) can inhibit DNMT1 in hepatocellular carcinoma (HCC). The aim of this study was to investigate the effect of 5-AZA-CdR in combination to and in comparison with SAHA on DNMT1, DNMT3a, DNMT3b, histone deacetylase 1 (HDAC1), glutathione S-transferase 1 (GSTP1) and suppressor of cytokine signaling 1 (SOCS1) genes expression, cell growth inhibition and apoptotic induction in hepatocellular LCL-PI 11 cell line.

**Materials and Methods: **The cells were treated with 5-AZA-CdR and SAHA and then MTT assay, cell apoptosis assay and Real-time quantitative RT-PCR (qRT-PCR) were done.

**Results: **Both agents indicated significant inhibitory and apoptotic effect (P< 0.001). The apoptotic effect of SAHA was more than that of 5-Aza-CdR. The result of qRT-PCR indicated that 5-Aza-CdR decreased DNMT1, DNMT3a, DNMT3b and increased GSTP1and SOCS1 genes expression and SAHA decreased HDAC1 and increased GSTP1 and SOCS1 genes expression significantly. Maximal apoptosis and genes expression were seen with combined treatment.

**Conclusion:** 5-AZA-CdR and SAHA down-regulated DNMT1, DNMT3a, DNMT3b, and HDAC1 and up-regulated GSTP1 and SOCS1 gene expression by which inhibited cell viability and induced apoptosis, suggesting that they could be used in the treatment of HCC.

## Introduction

 Aberrant methylation in the promoter-CpG islands of tumor suppressor genes (TSGs) is one of the most epigenetic alterations that involves in tumorigenesis and cancer progression. Several studies have reported that the methylation of TSGs such as SOCS-1, APC, E-cadherin, GSTP, p15, p16, RAR-, p14, and p73 genes leads to silenced genes resulting in HCC^1^. The DNA methylation process is catalyzed by DNA methyltransferases (DNMTs). The mammalian DNMTs are encoded by three distinct families of DNMT genes including DNMT1, DNMT2, and DNMT3. These enzymes catalyze the transfer of the methyl groups to DNA and methylate the CpG islands of TSGs. Increased expression of DNMT1, DNMT3a, and DNMT3b mRNA has been reported in HCC^2^. Additionally, histone deacetylation regulates chromatin remodeling and down-regulates TSGs in many types of cancer cells. Overexpression of histone deacetylase 1 (HDAC1) has been indicated in HCC^3^. The reversion of two epigenetic processes, hypermethylation and deacetylation by epi-drugs can restore normal expression of TSGs. It has been demonstrated that DNA mehyltransferase inhibitors such as 5-azacytidine (5-aza-CR, Vidaza), 5-aza-2′-deoxycytidine (5-aza-dC, Dacogen), and Zebularine (1-(β-_D_-ribofuranosyl)-1, 2-dihydropyrimidin-2-one) can restore hypermethylated TSGs resulting in apoptosis induction in several cancers, including HCC (HepG2, Hep3B, and Hepa1-6), renal, colon, and lung cancer cells ^4^^,^^5^. HDAC inhibitors (HDACIs) can induce cell-cycle arrest and cell apoptosis. Apoptotic effect of HDACIs such as butyrate, suberoylanilide hydroxamic acid (SAHA), depsipeptide, and MS-275 has been shown in various cancers such as colon cancer^6^, breast cancer, non–small-cell lung cancer (NSCLC), ovarian cancer^7^, and HCC (HepG2, MH1C1, Hepa1–6 and Hep1B)^8^. 

Previously, we reported that 5-aza-2′-deoxycytidine (5-AZA-CdR) and VPA can inhibit DNMT1 and induce apoptosis in HCC WCH-17 cell line ^9^. This result encouraged us to investigate the effect of 5-aza-2′-deoxycytidine (5-AZA-CdR) in combination with and in comparison to vorinostat (Suberoylanalide hydroxamic acid, SAHA) on DNMT1, DNMT3a, DNMT3b, histone deacetylase 1 (HDAC1) glutathione S-transferase 1 (GSTP1) and suppressor of cytokine signaling 1 (SOCS1) genes expression, cell growth inhibition and apoptotic induction in hepatocellular LCL-PI 11 cell line in the present study. 

## MATERIALS AND METHODS

**Materials **

Human HCC LCL-PI 11 cells were purchased from the National Cell Bank of Iran-Pasteur Institute and maintained in Dulbecco’s modified Eagle’s medium (DMEM), containing 100 mL/L fetal bovine serum and antibiotics at 37º C in a humidified atmosphere. 5-AZA-CdR and SAHA were purchased from Sigma (St. Louis, MO, USA) and dissolved in dimethyl sulfoxide (DMSO; Sigma) at a final concentration of 100 μM in order to prepare a working stock solution. All other necessary concentrations were provided by diluting this solution. 

Phosphate-buffered saline (PBS), 3-[4, 5-dimethyl-2-thiazolyl]-2, 5-diphenyl-2H-tetrazolium bromide (MTT), Trypsin-EDTA, DMSO, DMEM, Annexin-V-(FITC), propidium iodide (PI), streptomycin, and penicillin were purchased from Sigma. Real-time polymerase chain reaction (PCR) kits (qPCR MasterMix Plus for SYBR Green I dNTP) and total RNA extraction kit (TRIZOL reagent) were obtained from Applied Biosystems Inc. (Foster, CA, USA).

**Cell culture and cell viability**

The cells were seeded and cultured with DMEM (pH 7.2–7.4) supplemented with sodium pyruvate, sodium bicarbonate, 10% FBS, and antibiotics at 37°C in 5% CO2 overnight. Then, the cells were plated into 96-well plates at a density of 5 × 10^5 ^live cells per well. After one day, the cells were treated with medium, containing various concentrations of 5-Aza-CdR (1, 2.5, 5, 7.5 and 10 μM) and SAHA (1, 2.5, 5, 7.5 and 10 μM) for 24h and 48h. After treatment times, the effect of 5-Aza-CdR and SAHA was assessed by MTT assay according to

Standard protocols. In this regard, the culture medium was removed, the cells were washed twice with PBS and a fresh medium containing 0.5 mg/mL MTT was added. After 4 h incubation, the formazan crystals were dissolved in DMSO and the absorbance was measured by a microplate reader at a wavelength of 570 nM. Each experiment was repeated three times (triplicates).

**Cell apoptosis assay**

To assess the apoptotic cells by flow cytometry, the cells were plated (at a density of 5 × 10^5^) in 24-well and treated with 5-Aza-CdR (2.5 μM) and SAHA (2.5 μM) according to IC50, for 24 and 48 h. The control groups were incubated with DMSO only. After treatment, all the adherent cells were harvested by trypsinization and the single cell suspension was prepared. After centrifugation, the cells were washed with cold phosphate-buffered saline (PBS) and resuspended in Binding buffer (1x). For detection of apoptotic cells, annexin-V-(FITC) and propidium iodide (PI) were used according to the manufacturer's protocol. After incubation, the samples were subjected to flow cytometry and the apoptotic cells were counted by FACS can flow cytometry (Becton Dickinson, Heidelberg, Germany).

**Real-Time quantitative reverse transcription polymerase chain reaction**

**(qRT-PCR) analysis**

Real-time quantitative RT-PCR (qRT-PCR) was performed with the SYBR Green QuantiTect RT-PCR Kit (Qiagen) according to the manufacturer's instructions. To determine whether 5-Aza-CdR and SAHA could affect DNMT1, DNMT3a, DNMT3b, HDAC1, GSTP1and SOCS1 genes expression qRT-PCR was performed. The cells were treated with Aza-CdR and SAHA based on IC5o values, and total RNA was extracted using the RNeasy kit (Qiagen, Valencia, CA and treated by RNase‑free DNase (Qiagen) to eliminate the genomic DNA before cDNA synthesis. By using the RevertAid™ First Strand cDNA Synthesis Kit, total RNA was reverse transcribed to complementary DNA (cDNA) using oligodT primers and Superscript II Reverse Transcriptase according to the protocol. Real-time RT-PCR reactions for cDNA amplification were performed as mentioned previously^10^. The primer sequences are shown in Table 1. Quantitative RT-PCRs were performed using a Step One Real Time PCR System (Applied Biosystems). Thermal cycling conditions was : an initial denaturation at 95 ˚ C for 10 minutes, followed by 40 cycles of denaturation at 95˚ C for 20 seconds, annealing at 58 ˚c for 15 seconds and extension at 72 ˚ C for 15 seconds . GAPDH was used as an endogenous control. Data were analyzed using the comparative Ct (ΔΔct) method. 

The amount of target, normalized to the endogenous reference. The relative expression was calculated with the “delta-delta Ct method” (DDCt = DCtsample DCtcalibrator). The Ct (threshold cycle) values were normalized against the endogenous reference gene and were compared with the calibrator. Considering that DDCt validation required equal efficiencies of target genes and reference amplification, standard curve assays were obtained for target gene and reference.

**Table 1:** Primer sequences used in the present study

**Primer name**	**Primer sequences (5’ to 3’)**	**Reference**
DNMT1 Forward DNMT1 Reverse	GAG GAA GCT GCT AAG GAC TAG TTC ACT CCA CAA TTT GAT CAC TAA ATC	10
DNMT3a Forward DNMT3a Reverse	GGA GGC TGA GAA GAA AGC CAA GGT TTT GCC GTC TCC GAA CCA CAT GAC	10
DNMT3b Forward DNMT3b Reverse	TAC ACA GAC GTG TCC AAC ATG GGC GGA TGC CTT CAG GAA TCA CAC CTC	10
GSTP1 Forward GSTP1 Reverse	AGTTGCGCGGCGATTTC GCCCCAATACTAAATCACGACG	11
SOCS1 Forward SOCS1 Reverse	CGCGCGGGGTTTTCGTAGTA CTAACTCCAACCGTCCGACC	11
HDAC1 Forward HDAC1 Reverse	AGACAGCTGTGGCCCTGGATAC CGGCAGCATTCTAAGGTTCTCAA	12

**Statistical analysis**

The database was setup with the SPSS 16.0 software package (SPSS Inc., Chicago, Illinois, USA) for analysis. The data were acquired from three tests and are shown as means ± standard deviations. Statistical comparisons between groups were performed with ANOVA (one‑way ANOVA) and Turkey test. A significant difference was considered as P < 0.05.

## Results

**Result of cell viability by the MTT assay**

**Figure 1:**
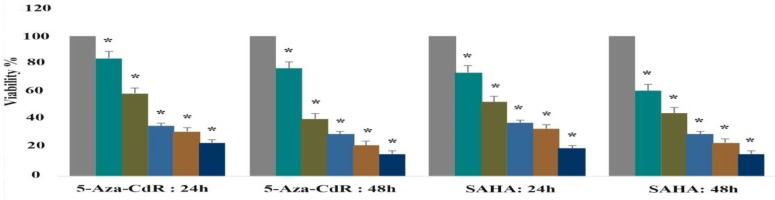
The effect of 5-AZA-CdR and SAHA on viability of LCL-PI 11.

 The LCL-PI 11 cells were treated with various doses of 5-Aza-CdR and SAHA for 24h and 48h as mentioned above, while control cells were received 0.05% DMSO. Then, the cell viability was determined by using the MTT assay. As shown in Figure 1, both agents indicated significant inhibitory effect with all concentrations (P< 0.001) in a dose and time-dependent manner. IC50 values were obtained with approximately 2.5 μM of 5-Aza-CdR and 2.5 μM of SAHA.

**Result of** **cell apoptosis assay**

**Figure 2:**
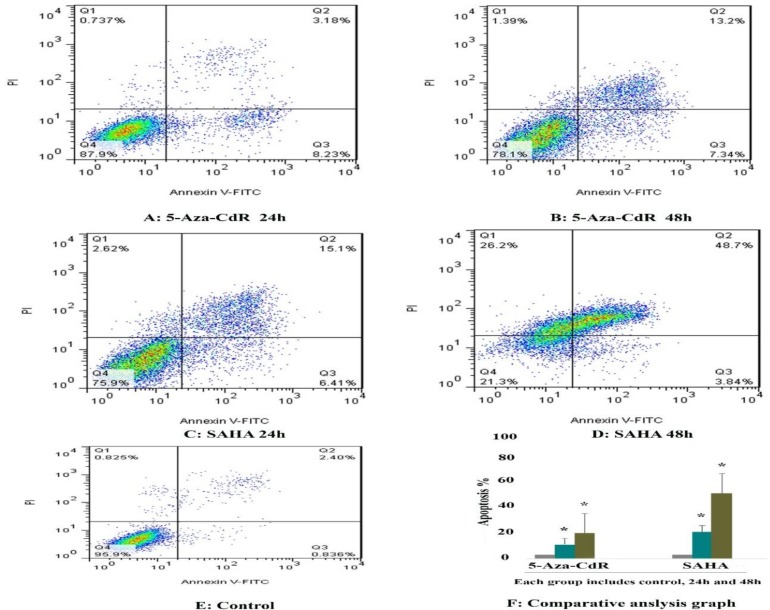
The apoptosis-inducing effect of 5-AZA-CdR and SAHA was investigated by flow cytometric analysis of HCC LCL-PI 11cells stained with Annexin V and propidium iodide. Result of flow cytometry indicated that both compounds induced cell apoptosis significantly. As indicated above, SAHA had more strong effect on apoptosis induction that than of SAHA. Asterisks (*) indicate significant differences between treated cells and the control groups.

**Figure 3:**
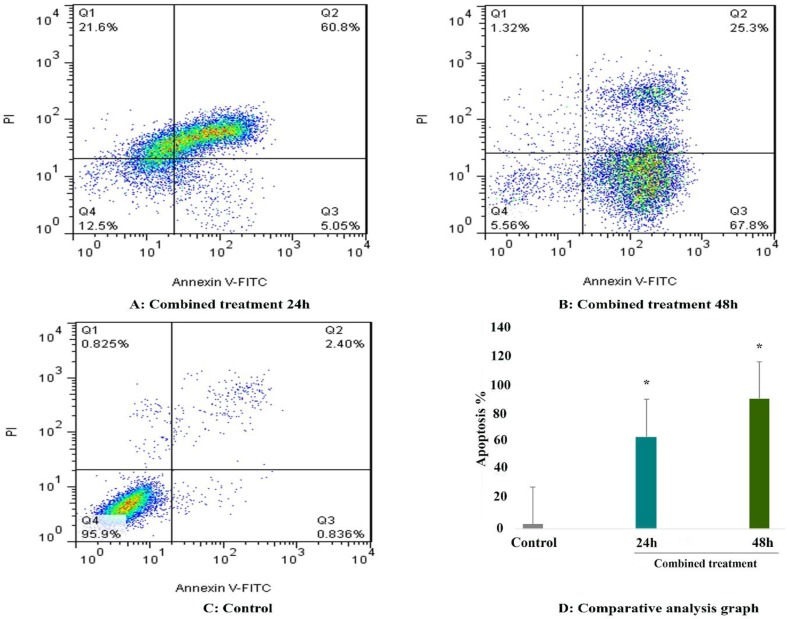
The apoptosis‑inducing effect of 5-AZA-CdR incombination to SAHA was investigated by flow cytometric analysis of HCC LCL-PI 11cells stained with Annexin V and propidium iodide. Result of flow cytometry indicated that combined treatment induced significant cell apoptosis more than each agent alone. Asterisks (*) indicate significant differences between treated cells and the control groups.

To determine whether 5-Aza-CdR and SAHA could induce apoptosis, we stained the cells using annexin-V-(FITC) and propidium iodide (PI). As mentioned above, the cells were treated with 5-Aza-CdR (2.5 μM) and SAHA (2.5 μM) for different time periods (24 and 48 h) and then collected after trypsinization and stained with Annexin V 

and propidium iodide following which investigated by flow cytometry. As shown in Figure 2, we observed a significant increase in the number of apoptotic cells in all treated groups. The percentage of apoptotic cells was 11.41 %, and 20.54 % after treatment with 5-Aza-CdR and 21% and 52.54 % after treatment with SAH and after 24 and 48h, respectively (P < 0.01). The apoptotic effect of SAHA was more than that of 5-Aza-CdR. Besides, maximal apoptosis was observed in the combined treatment groups as indicated in Figure 3 and Table 2.

**Result of determination of genes expression**

**Figure 4:**
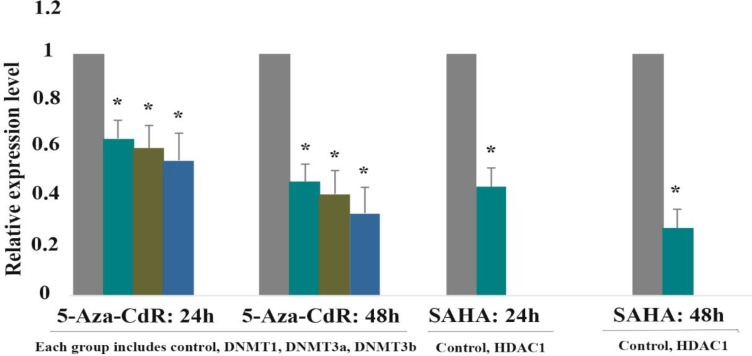
The relative expression level of DNMT1, DNMT3a, DNMT3b, and HDAC1 in response to 5-AZA-CdR and SAHA.

 The effect of 5-Aza-CdR (2.5 μM) and SAHA (2.5 μM) on DNMT1, DNMT3a, DNMT3b, HDAC1, GSTP1and SOCS1 genes expression was evaluated by quantitative real-time RT-PCR analysis at different time periods, including 24 and 48 h. The result indicated that 5-Aza-CdR decreased DNMT1, DNMT3a, DNMT3b and increased GSTP1and SOCS1 genes expression significantly. Besides, SAHA decreased HDAC1 and increased GSTP1 and SOCS1 genes expression significantly as demonstrated in Figure. 4 and Table 3. Meanwhile, SAHA had a more strong effect on the GSTP1and SOCS1 gene expression in comparison to 5-Aza-CdR. Additionally, maximal expression of GSTP1 and SOCS1 was seen with combined treatment.

The cells were treated without and with different doses of 5-AZA-CdR and SAHA for 24 and 48 h and the cell viability was evaluated by MTT assay. Each experiment was conducted in triplicate. Mean values from the three experiments ± standard error of mean are shown. First column of each group belongs control group. Asterisks (*) indicate significant differences between treated and untreated cells.

Quantitative reverse transcription polymerase chain reaction analysis demonstrated that 5-AZA-CdR decreased DNMT1, DNMT3a, and DNMT3b expression significantly. Similarly, SAHA induced significant down regulation in HDAC1. Asterisks (*) indicate significant differences between treated cells and the control group. Data are presented as means ± standard error of mean.

**Table 2:** The percentage of apoptotic cells treated with 5-Aza-CdR and SAHA.

**Drug**	**Dose (μM )**	**Duration (h)**	**Apoptosis (%)**	**P**
5-Aza-CdR	2.5	24	11.41	0.001
5-Aza-CdR	2.5	48	20.54	0.001
SAHA	2.5	24	21.51	0.001
SAHA	2.5	48	52.54	0.001
Combined	2.5/2.5	24	65.85	0.001
Combined	2.5/2.5	48	93.1	0.001

**Figure 5:**
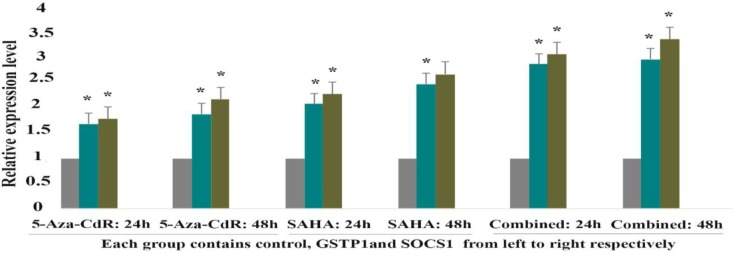
The relative expression level of GSTP1 AND SOCS1 in response to 5-AZA-CdR and SAHA (individually and combined).

The result indicated that both compounds (individually and combined) increased GSTP1 AND SOCS1 expression significantly. The combined treatment induced more up-regulation that than each agent individually. Asterisks (*) indicate significant differences between treated cells and the control group. Data are presented as means ± standard error of mean.

**Table 3:** The relative expression level of DNMT1, DNMT3a, DNMT3b, GSTP1and SOCS1 genes expression

**Gene**	**Drug**	**Dose (μM)**	**Duration (h)**	**Expression**	**P**
DNMT1	5-Aza-CdR	2.5 μM	24	0.65	0.011
DNMT3a	5-Aza-CdR	2.5 μM	24	0.61	0.005
DNMT3b	5-Aza-CdR	2.5 μM	24	0.56	0.002
DNMT1	5-Aza-CdR	2.5 μM	48	0.47	0.005
DNMT3a	5-Aza-CdR	2.5 μM	48	0.42	0.002
DNMT3b	5-Aza-CdR	2.5 μM	48	0.34	0.001
GSTP1	5-Aza-CdR	2.5 μM	24	1.7	0.001
GSTP1	5-Aza-CdR	2.5 μM	48	1.9	0.001
SCOS1	5-Aza-CdR	2.5 μM	24	1.8	0.001
SCOS1	5-Aza-CdR	2.5 μM	48	2.2	0.001
HDAC1	SAHA	2.5 μM	24	0.45	0.003
HDAC1	SAHA	2.5 μM	48	0.28	0.003
GSTP1	SAHA	2.5 μM	24	2.1	0.001
GSTP1	SAHA	2.5 μM	48	2.5	0.001
SCOS1	SAHA	2.5 μM	24	2.3	0.001
SCOS1	SAHA	2.5 μM	48	2.7	0.001
GSTP1	Combined	2.5 μM/2.5 μM	24	2.9	0.001
GSTP1	Combined	2.5 μM/2.5 μM	48	3	0.001
SCOS1	Combined	2.5 μM/2.5 μM	24	3.1	0.001
SCOS1	Combined	2.5 μM/2.5 μM	48	3.4	0.001

## Discussion

 Several TSGs have been demonstrated to be silenced in human cancers. In fact, the silencing is accompanied by epigenetic alterations of histone deacetylation and DNA hypermethylation of the promoter regions of TSGs. Aberrant DNA methylation and histone deacetylation of these genes have been reported in HCC ^13^^, ^^14^. Fortunately, epigenetic alterations are reversible and could be modified in response to DNMT and HDAC inhibitors. Because histone deacetylation and DNA methylation are the two best-characterized covalent modifications associated with chromatin compaction and silenced TSGs, we focused our attention on these changes. In the present study, we indicated that DNMT inhibitor 5-AZA-CdR and histone deacetylase inhibitor SAHA re-activate GSTP1 and SOCS1 gene expression epigenetically. We reported that these compounds up-regulate GSTP1 and SOCS1 gene expression by down-regulation of DNMT1, DNMT3a, DNMT3b, and HDAC1, resulting in cell growth inhibition and apoptosis induction. Furthermore, we demonstrated that SAHA had a more strong effect on GSTP1 and SOCS1 gene expression than that of 5-AZA-CdR. Besides, maximal GSTP1 and SOCS1 genes expression and apoptotic induction were seen after combined treatment. Consistent with our findings, hypermethylation of numerous TSGs have been reported in HCC, including GSTP1, SOCS-1, RASSF1A, APC, p16, MGMT, DAPK, and RIZ1 ^15^. Another study has been indicated that GSTP1, CCND2, RASSF1A, SPINT2, APC, RUNX3, and CFTR are aberrantly methylated in HCC ^16^. There are similar reports about p16 (INK4a), p15 (INK4b) ^17^, GSTP1 in HCC Hep3B, HepG2 ^18^, and GSTP1 in HCC Hep3B^19^. In addition to hypermethylation, we indicated that HDAC1 activity is one of the most characterized causes of HCC. In line with our findings, high HDAC1 expression has been shown in this cancer^20^. Other researchers have been reported overexpression of various HDACs in HCC, including HDAC2 in Hep3B cells^21^, HDAC5^22^, HDAC6 ^23^^, ^^24^, and HDAC8 ^25^. As we indicated in this study, several lines of evidence have been reported that 5-AZA-CdR can re-express GSTP1 by demethylation in prostate cancer^26^ and gastric cancer ^27^. In addition to 5-AZA-CdR, other DNMTIs can re-activate GSTP1 in prostate cell lines (PC-3, DU-145, LNCaP)^28^. Similarly, it has been shown that 5-AZA-CdR can re-activate SOCS1 expression in breast cancer cells^29^. We showed that SAHA up-regulated GSTP1 and SOCS1 through HDAC1 down-regulation. Consistent with this observation**, **another study demonstrated that SAHA inhibits HDAC1 by which increases p19^INK4d^ and p21^Waf1/Cip1^ expression and apoptosis induction in HCC ^30^. Similar inhibition has been reported in MEL cells ^31^. We observed maximal expression of GSTP1 and SOCS1 after combined treatment with 5-AZA-CdR and SAHA. Consistent with our observation, it has been concluded that the combination therapy of DNMTIs and histone deacetylase inhibitor shows synergistic anticancer effect^32^. Other researchers have been reported that GE in combination with trichostatin A (TSA) significantly increases TNFR-1 gene expression and apoptosis induction in lung cancer A549 cell line^33^. Previously, we demonstrated that GE and TSA can significantly reactivate the estrogen receptor alpha (ERα) gene and play a significant role in apoptosis induction in HCC ^34^. With regard to our previous results and the results of other researchers mentioned above, it is obvious that none of the studies have shown the effect of 5-AZA-CdR and SAHA (individually and combined) on GSTP1 and SOCS1 gene expression as a mechanism of apoptosis induction. Therefore, it is a novelty of our work. It should be noted that the effect of 5-AZA-CdR on GSTP1 and SOCS1 re-activation is one of the several mechanisms of this agent on apoptosis induction. Other pathways include down-regulation of anti-apoptotic XIAP, cIAP-1, Bcl-2, and cIAP-2 protein levels, the activation of caspases, the cleavage of Bid proteins, and the collapse of mitochondrial membrane potential (MMP) ^35^. Besides, there are many mechanisms of the antiapoptotic effect of HDACIs in addition to HDAC1 inhibition. The mechanisms by which these compounds induce apoptosis involving the accumulation of acetylated forms of histones and non‐histone protein substrates.

There is evidence that indicates that certain HDACIs may selectively inhibit different HDACs (e.g. TSA is a potent inhibitor of HDACs 1, 3, and 8)^36^. Although, we reported two epigenetic causes of HCC including hypermethylation and deacetylation of TSGs GSTP1 and SOCS1 we didn’t evaluate protein expression status of these genes in the current study. Therefore, protein expression evaluation is recommended.

## CONCLUSION

 5-AZA-CdR and SAHA down-regulated DNMT1, DNMT3a, DNMT3b, and HDAC1 and up-regulated GSTP1 and SOCS1 gene expression by which inhibited cell viability and induced apoptosis, suggesting that they could be used in the treatment of HCC. 

## References

[1] Yang B, Guo M, Herman JG (2003). Aberrant promoter methylation profiles of tumor suppressor genes in hepatocellular carcinoma. Am J Pathol.

[2] Oh BK, Kim H, Park HJ (2007). DNA methyltransferase expression and DNA methylation in human hepatocellular carcinoma and their clinicopathological correlation. Int J Mol Med.

[3] Buurman R, Gürlevik E, Schäffer V (2012). Histone deacetylases activate hepatocyte growth factor signaling by repressing microRNA-449 in hepatocellular carcinoma cells. Gastroenterology.

[4] Hong YK, Li Y, Pandit H (2019). Epigenetic modulation enhances immunotherapy for hepatocellular carcinoma. Cell Immunol..

[5] Venturelli S, Berger A, Weiland T (2013). Differential induction of apoptosis and senescence by the DNA methyltransferase inhibitors 5-azacytidine and 5-aza-2′-deoxycytidine in solid tumor cells. Mol Cancer Ther.

[6] Johnstone RW, Licht JD (2003). Histone deacetylase inhibitors in cancer therapy: is transcription the primary target?. Cancer Cell.

[7] Lane AA, Chabner BA (2009). Histone deacetylase inhibitors in cancer therapy. J Clin Oncol.

[8] Herold C, Ganslmayer M, Ocker M (2002). The histone-deacetylase inhibitor Trichostatin A blocks proliferation and triggers apoptotic programs in hepatoma cells. J Hepatol.

[9] Sanaei M, Kavoosi F (2019). Effects of 5-aza-2ˈ-deoxycytidine and Valproic Acid on Epigenetic modifying DNMT1 Gene Expression, Apoptosis Induction and Cell Viability in Hepatocellular Carcinoma WCH-17 cell line. Iran J Ped Hematol Oncol.

[10] Sanaei M, Kavoosi F, Roustazadeh A (2018). Effect of genistein in comparison with trichostatin a on reactivation of DNMTs genes in hepatocellular carcinoma. J Clin Transl Hepatol.

[11] Nomoto S, Kinoshita T, Kato K (2007). Hypermethylation of multiple genes as clonal markers in multicentric hepatocellular carcinoma. Br J Cancer.

[12] Kawabata T, Nishida K, Takasugi K (2010). Increased activity and expression of histone deacetylase 1 in relation to tumor necrosis factor-alpha in synovial tissue of rheumatoid arthritis. Arthritis Res Ther.

[13] Kondo Y, Shen L, Suzuki S (2007). Alterations of DNA methylation and histone modifications contribute to gene silencing in hepatocellular carcinomas. Hepatol Res.

[14] Zhang C, Li H, Wang Y (2010). Epigenetic inactivation of the tumor suppressor gene RIZ1 in hepatocellular carcinoma involves both DNA methylation and histone modifications. J Hepatol.

[15] Lou C, Yang B, Gao YT (2008). Aberrant methylation of multiple genes and its clinical implication in hepatocellular carcinoma. Zhonghua zhong liu za zhi.

[16] Moribe T, Iizuka N, Miura T (2009). Methylation of multiple genes as molecular markers for diagnosis of a small, well‐differentiated hepatocellular carcinoma. Int J Cancer.

[17] Dong Y, Wang A (2014). Aberrant DNA methylation in hepatocellular carcinoma tumor suppression. Oncol Lett.

[18] Zhong S, Tang MW, Yeo W (2002). Silencing of GSTP1 gene by CpG island DNA hypermethylation in HBV-associated hepatocellular carcinomas. Clin Cancer Res.

[19] Tchou JC, Lin X, Freije D (2000). GSTP1 CpG island DNA hypermethylation in hepatocellular carcinomas. Int J Oncol.

[20] Rikimaru T, Taketomi A, Yamashita Y (2007). Clinical significance of histone deacetylase 1 expression in patients with hepatocellular carcinoma. Oncology.

[21] Noh JH, Jung KH, Kim JK (2011). Aberrant regulation of HDAC2 mediates proliferation of hepatocellular carcinoma cells by deregulating expression of G1/S cell cycle proteins. PLoS One.

[22] Feng GW, Dong LD, Shang WJ (2014). HDAC5 promotes cell proliferation in human hepatocellular carcinoma by up-regulating Six1 expression. Eur Rev Med Pharmacol Sci.

[23] Ding G, Liu HD, Huang Q (2013). HDAC6 promotes hepatocellular carcinoma progression by inhibiting P53 transcriptional activity. FEBS Lett.

[24] Kanno K, Kanno S, Nitta H (2012). Overexpression of histone deacetylase 6 contributes to accelerated migration and invasion activity of hepatocellular carcinoma cells. Oncol Rep.

[25] Wu J, Du C, Lv Z (2013). The up-regulation of histone deacetylase 8 promotes proliferation and inhibits apoptosis in hepatocellular carcinoma. Dig Dis Sci.

[26] Chiam K, Centenera MM, Butler LM (2011). GSTP1 DNA methylation and expression status is indicative of 5-aza-2′-deoxycytidine efficacy in human prostate cancer cells. PLoS One.

[27] Vardi A, Bosviel R, Rabiau N (2010). Soy phytoestrogens modify DNA methylation of GSTP1, RASSF1A, EPH2 and BRCA1 promoter in prostate cancer cells. In Vivo.

[28] To KF, Chan MW, Leung WK (2004). Constitutional activation of IL-6-mediated JAK/STAT pathway through hypermethylation of SOCS-1 in human gastric cancer cell line. Br J Cancer.

[29] Evans MK, Yu CR, Lohani A (2007). Expression of SOCS1 and SOCS3 genes is differentially regulated in breast cancer cells in response to proinflammatory cytokine and growth factor signals. Oncogene.

[30] Zhou H, Cai Y, Liu D (2018). Pharmacological or transcriptional inhibition of both HDAC 1 and 2 leads to cell cycle blockage and apoptosis via p21Waf1/Cip1 and p19INK4d upregulation in hepatocellular carcinoma. Cell Prolif.

[31] Huang L, Pardee AB (2000). Suberoylanilide hydroxamic acid as a potential therapeutic agent for human breast cancer treatment. Mol Med.

[32] Roh MS, Kim CW, Park BS (2004). Mechanism of histone deacetylase inhibitor Trichostatin A induced apoptosis in human osteosarcoma cells. Apoptosis.

[33] Wu TC, Yang YC, Huang PR (2012). Genistein enhances the effect of trichostatin A on inhibition of A549 cell growth by increasing expression of TNF receptor-1. Toxicol Appl Pharmacol.

[34] Sanaei M, Kavoosi F, Salehi H (2017). Genistein and trichostatin A induction of estrogen receptor alpha gene expression, apoptosis and cell growth inhibition in hepatocellular carcinoma HepG 2 cells. Asian Pac J Cancer Prev.

[35] Shin DY, Park YS, Yang K (2012). Decitabine, a DNA methyltransferase inhibitor, induces apoptosis in human leukemia cells through intracellular reactive oxygen species generation. Int J Oncol.

[36] Dokmanovic M, Marks PA (2005). Prospects: histone deacetylase inhibitors. J Cell Biochem.

